# Diammonium tricadmium tris­(sulfate) dihydroxide dihydrate

**DOI:** 10.1107/S1600536811012979

**Published:** 2011-04-13

**Authors:** Xin Yin

**Affiliations:** a Department of Chemistry, East China University of Science and Technology, School of Chemistry and Molecular Engineering, Mei Long Road 130, Shanghai 200237, People’s Republic of China

## Abstract

The title compound, (NH_4_)_2_Cd_3_(SO_4_)_3_(OH)_2_(H_2_O)_2_, has been obtained serendipitously. It is isotypic with the heavier alkali analogues *M*_2_Cd_3_(SO_4_)_3_(OH)_2_(H_2_O)_2_ (*M* = K, Rb, Cs). The structure contains two Cd^2+^ ions, one in a general position and one with site symmetry *m*. The former Cd^2+^ ion is coordinated by three O atoms of three SO_4_ groups, two hydroxide O atoms and one water O atom, the latter Cd^2+^ ion by four O atoms of four SO_4_ groups and two hydroxide O atoms, both in a distorted octa­hedral coordination geometry. This arrangement leads to the formation of a layered framework extending parallel to (100), with the ammonium cations situated in the voids. O—H⋯O hydrogen bonds involving the water mol­ecules, hydroxide groups and sulfate O atoms, as well as N—H⋯O hydrogen bonds between ammonium cations and sulfate O atoms consolidate the crystal packing.

## Related literature

For the isotypic K and Cs analogues, see: Louer & Louer (1982 ▶), and for the Rb analogue, see: Swain & Guru Row (2006 ▶).

## Experimental

### 

#### Crystal data

(NH_4_)_2_Cd_3_(SO_4_)_3_(OH)_2_(H_2_O)_2_
*M* *_r_* = 731.51
Orthorhombic, 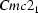

*a* = 18.906 (3) Å
*b* = 7.9483 (11) Å
*c* = 9.9809 (13) Å
*V* = 1499.8 (4) Å^3^
*Z* = 4
Mo *K*α radiation
μ = 4.72 mm^−1^
*T* = 296 K
0.12 × 0.10 × 0.08 mm

#### Data collection

Bruker SMART CCD diffractometer
Absorption correction: multi-scan (*SADABS*; Sheldrick, 2003 ▶) *T*_min_ = 0.601, *T*_max_ = 0.704
7060 measured reflections
1770 independent reflections
1739 reflections with *I* > 2σ(*I*)
*R*_int_ = 0.051

#### Refinement

*R*[*F* ^2^ > 2σ(*F* ^2^)] = 0.027
*wR*(*F* ^2^) = 0.066
*S* = 1.08
1770 reflections
118 parameters
1 restraint
H-atom parameters constrained
Δρ_max_ = 0.68 e Å^−3^
Δρ_min_ = −1.31 e Å^−3^
Absolute structure: Flack (1983 ▶), 825 Friedel pairs
Flack parameter: −0.07 (4)

### 

Data collection: *SMART* (Bruker, 2001 ▶); cell refinement: *SAINT-Plus* (Bruker, 2003 ▶); data reduction: *SAINT-Plus*; program(s) used to solve structure: *SHELXTL* (Sheldrick, 2008 ▶); program(s) used to refine structure: *SHELXTL*; molecular graphics: *Mercury* (Macrae *et al.*, 2006 ▶) and *DIAMOND* (Brandenburg, 2006 ▶); software used to prepare material for publication: *SHELXTL*.

## Supplementary Material

Crystal structure: contains datablocks I, global. DOI: 10.1107/S1600536811012979/wm2470sup1.cif

Structure factors: contains datablocks I. DOI: 10.1107/S1600536811012979/wm2470Isup2.hkl

Additional supplementary materials:  crystallographic information; 3D view; checkCIF report

## Figures and Tables

**Table 1:** Hydrogen-bond geometry (Å, °)

*D*—H⋯*A*	*D*—H	H⋯*A*	*D*⋯*A*	*D*—H⋯*A*
O10—H10*B*⋯O7^i^	0.85	2.25	3.087 (5)	167
O10—H10*B*⋯O5^ii^	0.85	2.36	2.985 (6)	131
O9—H9*A*⋯O6^iii^	0.85	2.18	3.029 (7)	173
O8—H8*A*⋯O5^ii^	0.85	2.56	3.322 (9)	150
O8—H8*A*⋯O5^i^	0.85	2.56	3.322 (9)	150
N1—H1*B*⋯O10^iv^	0.90	2.26	2.948 (6)	133
N1—H1*D*⋯O4^ii^	0.90	2.18	3.077 (5)	180
N1—H1*C*⋯O4^v^	0.90	2.19	3.074 (7)	168
N1—H1*A*⋯O3^vi^	0.90	2.38	2.995 (6)	126
N1—H1*D*⋯O2^ii^	0.90	2.64	3.196 (7)	121

Symmetry codes: (i) 
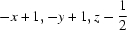
; (ii) 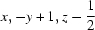; (iii) 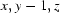; (iv) 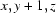; (v) 
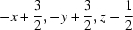
; (vi) 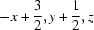.

**Fig. 1.**
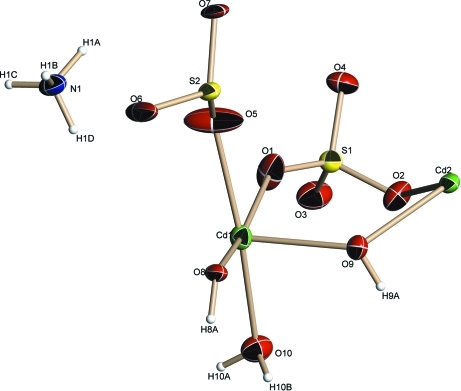
The asymmetric unit of (I), with displacement parameters shown at the 30% probability level.

**Fig. 2.**
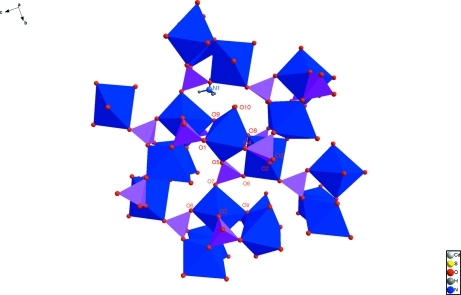
View of the two-dimensional network structure of (I) parallel to (100) in the polyhedral representation.

**Fig. 3.**
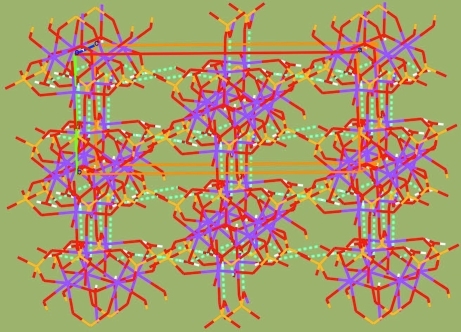
Three-dimensional supramolecular structure of (I), built up through hydrogen bonding. NH4+ ions have been omitted for clarity.

## supplementary crystallographic information

### Comment

The title compound, (NH_4_)_2_Cd_3_(SO_4_)_3_(OH)_2_(H_2_O)_2_, (I), formed accidentally under hydrothermal reaction conditions. Our intended target product was to synthesis a coordination compound from 1*H*-benzimidazole-5,6-dicarboxylate and CdSO_4_^.^8/3H_2_O. The presence of ammonium ions in the finally obtained compound points to an internal redox process that presumably has caused a (partly) reduction of the nitrogen atoms of 1*H*-benzimidazole-5,6-dicarboxylate or the nitrate anions. (NH_4_)_2_Cd_3_(SO_4_)_3_(OH)_2_(H_2_O)_2_ is isotypic with other *M*_2_Cd_3_(SO_4_)_3_(OH)_2_(H_2_O)_2_ members (*M* = K, Cs (Louer & Louer, 1982); *M* = Rb (Swain & Guru Row, 2006)).

The asymmetric unit of (I) is illustrated in Fig. 1. The crystal structure of (NH_4_)_2_Cd_3_(SO_4_)_3_(OH)_2_(H_2_O)_2_ is made up from two different Cd^2+^ ions (one on a general position (Cd1), one with site symmetry *m* (Cd2)), two sulfate ions (likewise one on a general position and the other with site symmetry *m*), two hydroxide groups, one water molecule and one NH_4_^+^ cation. Both Cd^2+^ cations are six-coordinated in an octahedral coordination geometry. Cd2 is coordinated by four sulfate ions and two hydroxide ions, while Cd1 is coordinated by three sulfate ons, two hydroxide anions and one water molecule. There are four types of oxygen atoms in the crystal structure of the title compound. The O3 atom of one SO_4_^2-^ anion is solely bound to the S atom, O8 and O9 represent the oxygen atoms of hydroxide groups shared by three Cd atoms, O10 is the water O atom bound to one Cd atom and all other O atoms represent sulfate O atoms coordinated to only one Cd atom.

As can be seen in Fig. 2, the Cd(1)O_6_ polyhedra are connected by sharing edges of OH groups. Cd(2)O_6_ octahedra and SO_4_ tetrahedra are linked to these dimers *via* common corners, thus forming a two-dimensional network extending parallel to the *bc* plane. The NH_4_^+^ cations are situated in the voids of the layers. Through formation of N—H···O and O—H···O hydrogen bonds a three-dimensional structure is formed. Since all water and hydroxide groups and most of the sulfate O atoms are involved in hydrogen bonding, the resulting network can be considered as relatively stable (Table 2, Fig. 3).

### Experimental

All reagents were obtained from commercial sources and used without further purification. A mixture of CdSO_4_^.^8/3H_2_O (0.2565 g, 1.0 mmol), 1*H*-benzimidazole-5,6-dicarboxylate (0.1236 g,0.6 mmol), CH_3_CN (6 ml) and 4 ml water were added to a 23 ml Teflon-lined stainless container, which was heated to 423 K and held at that temperature for 5 days. After cooling to room temperature in 24 h, colourless crystals were recovered by filtration (yield 49% based on CdSO_4_^.^8/3H_2_O).

### Refinement

The H atoms were localized from a difference Fourier map. Their coordinates were refined independently with O—H distances restrained to 0.85 (2) Å and the H—H = 1.30 (2)Å for the water H atoms. The isotropic temperature parameters of the H atoms were refined with 1.2*U*eq of the parent atom. H atoms of the ammonium cation were placed in calculated positions, with N—H = 0.90Å, *Uiso*(H) = 1.2 *Ueq*(N). The deepest hole in the final Fourier map is 0.8 Å from Cd2.

### Crystal data

**Table:**

Cd_3_H_6_O_16_S_3_·2NH_4_	*F*(000) = 1392
*M**_r_* = 731.51	*D*_x_ = 3.240 Mg m^−^^3^
Orthorhombic, *C**m**c*2_1_	Mo *K*α radiation, λ = 0.71073 Å
Hall symbol: C 2c -2	Cell parameters from 12815 reflections
*a* = 18.906 (3) Å	θ = 1.7–27.5°
*b* = 7.9483 (11) Å	µ = 4.72 mm^−^^1^
*c* = 9.9809 (13) Å	*T* = 296 K
*V* = 1499.8 (4) Å^3^	Block, colourless
*Z* = 4	0.12 × 0.10 × 0.08 mm

### Data collection

**Table:**

Bruker SMART CCD diffractometer	1770 independent reflections
Radiation source: fine-focus sealed tube	1739 reflections with *I* > 2σ(*I*)
graphite	*R*_int_ = 0.051
φ and ω–scans	θ_max_ = 27.5°, θ_min_ = 3.5°
Absorption correction: multi-scan (*SADABS*; Sheldrick, 2003)	*h* = −24→24
*T*_min_ = 0.601, *T*_max_ = 0.704	*k* = −10→10
7060 measured reflections	*l* = −12→12

### Refinement

**Table:**

Refinement on *F*^2^	Secondary atom site location: difference Fourier map
Least-squares matrix: full	Hydrogen site location: inferred from neighbouring sites
*R*[*F*^2^ > 2σ(*F*^2^)] = 0.027	H-atom parameters constrained
*wR*(*F*^2^) = 0.066	*w* = 1/[σ^2^(*F*_o_^2^) + (0.0328*P*)^2^ + 1.1583*P*] where *P* = (*F*_o_^2^ + 2*F*_c_^2^)/3
*S* = 1.08	(Δ/σ)_max_ < 0.001
1770 reflections	Δρ_max_ = 0.68 e Å^−^^3^
118 parameters	Δρ_min_ = −1.31 e Å^−^^3^
1 restraint	Absolute structure: Flack (1983), 825 Friedel pairs
Primary atom site location: structure-invariant direct methods	Flack parameter: −0.07 (4)

### Special details

**Table:**

Geometry. All esds (except the esd in the dihedral angle between two l.s. planes) are estimated using the full covariance matrix. The cell esds are taken into account individually in the estimation of esds in distances, angles and torsion angles; correlations between esds in cell parameters are only used when they are defined by crystal symmetry. An approximate (isotropic) treatment of cell esds is used for estimating esds involving l.s. planes.
Refinement. Refinement of *F*^2^ against ALL reflections. The weighted *R*-factor *wR* and goodness of fit *S* are based on *F*^2^, conventional *R*-factors *R* are based on *F*, with *F* set to zero for negative *F*^2^. The threshold expression of *F*^2^ > σ(*F*^2^) is used only for calculating *R*-factors(gt) *etc*. and is not relevant to the choice of reflections for refinement. *R*-factors based on *F*^2^ are statistically about twice as large as those based on *F*, and *R*- factors based on ALL data will be even larger.

### Fractional atomic coordinates and isotropic or equivalent isotropic displacement parameters (Å^2^)

**Table:**

	*x*	*y*	*z*	*U*_iso_*/*U*_eq_	
Cd1	0.588435 (18)	0.46065 (4)	0.29502 (5)	0.01981 (11)	
Cd2	0.5000	0.19102 (5)	0.58110 (4)	0.01744 (12)	
S1	0.67635 (6)	0.30002 (13)	0.57340 (14)	0.0184 (2)	
S2	0.5000	0.8103 (2)	0.41513 (16)	0.0192 (4)	
O1	0.6589 (3)	0.4252 (6)	0.4718 (4)	0.0387 (11)	
O2	0.6223 (2)	0.1661 (4)	0.5730 (5)	0.0299 (8)	
O3	0.7446 (2)	0.2229 (5)	0.5454 (5)	0.0357 (11)	
O4	0.6794 (2)	0.3814 (5)	0.7053 (4)	0.0299 (9)	
O5	0.5621 (3)	0.7036 (8)	0.4165 (6)	0.0583 (18)	
O6	0.5000	0.9205 (6)	0.3000 (7)	0.0500 (19)	
O7	0.5000	0.9098 (6)	0.5395 (5)	0.0303 (13)	
O8	0.5000	0.5445 (5)	0.1571 (5)	0.0192 (11)	
H8A	0.5000	0.4922	0.0828	0.029*	
O9	0.5000	0.2910 (6)	0.3712 (5)	0.0201 (10)	
H9A	0.5000	0.1896	0.3440	0.030*	
O10	0.6329 (2)	0.2327 (5)	0.1789 (4)	0.0293 (8)	
H10A	0.6695	0.2468	0.1308	0.044*	
H10B	0.5985	0.2025	0.1297	0.044*	
N1	0.6921 (3)	0.9615 (4)	0.3464 (5)	0.0238 (10)	
H1A	0.6950	0.9457	0.4356	0.029*	
H1B	0.6538	1.0244	0.3276	0.029*	
H1C	0.7312	1.0147	0.3174	0.029*	
H1D	0.6883	0.8612	0.3052	0.029*	

### Atomic displacement parameters (Å^2^)

**Table:**

	*U*^11^	*U*^22^	*U*^33^	*U*^12^	*U*^13^	*U*^23^
Cd1	0.02190 (18)	0.01799 (18)	0.01954 (18)	0.00123 (11)	−0.00023 (14)	0.00305 (13)
Cd2	0.0246 (3)	0.0116 (2)	0.0161 (2)	0.000	0.000	−0.00027 (18)
S1	0.0206 (6)	0.0174 (5)	0.0172 (5)	0.0020 (4)	−0.0014 (5)	0.0002 (4)
S2	0.0299 (10)	0.0120 (7)	0.0157 (8)	0.000	0.000	−0.0020 (5)
O1	0.051 (3)	0.034 (2)	0.032 (2)	−0.014 (2)	−0.024 (2)	0.0156 (18)
O2	0.028 (2)	0.0217 (15)	0.040 (2)	−0.0028 (14)	−0.0029 (18)	−0.0014 (18)
O3	0.027 (2)	0.036 (3)	0.045 (2)	0.0036 (19)	0.0040 (16)	−0.0123 (17)
O4	0.036 (2)	0.034 (2)	0.0201 (17)	0.0036 (18)	−0.0002 (15)	−0.0073 (15)
O5	0.059 (4)	0.066 (3)	0.051 (3)	0.042 (3)	−0.027 (3)	−0.034 (3)
O6	0.120 (6)	0.016 (2)	0.014 (2)	0.000	0.000	−0.001 (3)
O7	0.063 (4)	0.011 (2)	0.016 (2)	0.000	0.000	−0.0070 (19)
O8	0.031 (3)	0.014 (2)	0.012 (2)	0.000	0.000	−0.0018 (16)
O9	0.023 (3)	0.016 (2)	0.021 (2)	0.000	0.000	0.0034 (17)
O10	0.024 (2)	0.0293 (19)	0.034 (2)	0.0021 (16)	−0.0009 (16)	−0.0066 (17)
N1	0.034 (3)	0.016 (2)	0.022 (2)	0.0022 (15)	−0.0038 (19)	−0.0032 (17)

### Geometric parameters (Å, °)

**Table:**

Cd1—O1	2.228 (4)	S2—O6	1.444 (6)
Cd1—O8	2.266 (3)	S2—O5^ii^	1.449 (5)
Cd1—O9	2.278 (3)	S2—O5	1.449 (5)
Cd1—O10	2.309 (4)	S2—O7	1.472 (5)
Cd1—O4^i^	2.310 (4)	O4—Cd1^v^	2.310 (4)
Cd1—O5	2.333 (5)	O6—Cd2^vi^	2.358 (6)
Cd1—Cd1^ii^	3.3439 (8)	O7—Cd2^vii^	2.274 (5)
Cd2—O8^iii^	2.235 (4)	O8—Cd2^vi^	2.235 (4)
Cd2—O9	2.241 (5)	O8—Cd1^ii^	2.266 (3)
Cd2—O7^iv^	2.274 (5)	O8—H8A	0.8500
Cd2—O2	2.323 (4)	O9—Cd1^ii^	2.278 (3)
Cd2—O2^ii^	2.323 (4)	O9—H9A	0.8501
Cd2—O6^iii^	2.358 (6)	O10—H10A	0.8500
Cd2—H9A	2.3665	O10—H10B	0.8501
S1—O3	1.456 (4)	N1—H1A	0.9000
S1—O1	1.459 (4)	N1—H1B	0.9001
S1—O4	1.468 (4)	N1—H1C	0.9001
S1—O2	1.475 (4)	N1—H1D	0.9000
			
O1—Cd1—O8	163.57 (18)	O2—Cd2—Cd1^iii^	120.64 (10)
O1—Cd1—O9	95.73 (17)	O2^ii^—Cd2—Cd1^iii^	69.55 (10)
O8—Cd1—O9	80.52 (14)	O6^iii^—Cd2—Cd1^iii^	76.00 (12)
O1—Cd1—O10	94.62 (17)	Cd1^v^—Cd2—Cd1^iii^	51.115 (14)
O8—Cd1—O10	101.21 (15)	O8^iii^—Cd2—H9A	110.1
O9—Cd1—O10	88.27 (16)	O9—Cd2—H9A	21.0
O1—Cd1—O4^i^	86.03 (15)	O7^iv^—Cd2—H9A	79.2
O8—Cd1—O4^i^	98.85 (13)	O2—Cd2—H9A	88.0
O9—Cd1—O4^i^	175.79 (16)	O2^ii^—Cd2—H9A	88.0
O10—Cd1—O4^i^	87.77 (15)	O6^iii^—Cd2—H9A	157.7
O1—Cd1—O5	79.68 (19)	Cd1^v^—Cd2—H9A	123.7
O8—Cd1—O5	85.11 (16)	Cd1^iii^—Cd2—H9A	123.7
O9—Cd1—O5	99.2 (2)	O3—S1—O1	110.7 (3)
O10—Cd1—O5	171.0 (2)	O3—S1—O4	108.8 (3)
O4^i^—Cd1—O5	84.9 (2)	O1—S1—O4	109.4 (3)
O1—Cd1—Cd1^ii^	126.71 (14)	O3—S1—O2	108.0 (2)
O8—Cd1—Cd1^ii^	42.45 (9)	O1—S1—O2	109.5 (3)
O9—Cd1—Cd1^ii^	42.79 (8)	O4—S1—O2	110.3 (3)
O10—Cd1—Cd1^ii^	111.34 (10)	O6—S2—O5^ii^	111.2 (3)
O4^i^—Cd1—Cd1^ii^	138.11 (10)	O6—S2—O5	111.2 (3)
O5—Cd1—Cd1^ii^	77.68 (16)	O5^ii^—S2—O5	108.3 (6)
O1—Cd1—Cd2^vi^	141.66 (12)	O6—S2—O7	110.2 (3)
O8—Cd1—Cd2^vi^	30.33 (10)	O5^ii^—S2—O7	107.9 (2)
O9—Cd1—Cd2^vi^	106.86 (9)	O5—S2—O7	107.9 (2)
O10—Cd1—Cd2^vi^	116.18 (10)	S1—O1—Cd1	140.5 (3)
O4^i^—Cd1—Cd2^vi^	73.69 (10)	S1—O2—Cd2	128.9 (2)
O5—Cd1—Cd2^vi^	66.61 (14)	S1—O4—Cd1^v^	123.9 (2)
Cd1^ii^—Cd1—Cd2^vi^	64.443 (7)	S2—O5—Cd1	130.8 (3)
O8^iii^—Cd2—O9	89.07 (18)	S2—O6—Cd2^vi^	120.6 (3)
O8^iii^—Cd2—O7^iv^	170.68 (18)	S2—O7—Cd2^vii^	133.0 (3)
O9—Cd2—O7^iv^	100.25 (17)	Cd2^vi^—O8—Cd1^ii^	118.87 (13)
O8^iii^—Cd2—O2	95.29 (9)	Cd2^vi^—O8—Cd1	118.87 (13)
O9—Cd2—O2	89.88 (12)	Cd1^ii^—O8—Cd1	95.09 (18)
O7^iv^—Cd2—O2	84.82 (8)	Cd2^vi^—O8—H8A	99.4
O8^iii^—Cd2—O2^ii^	95.29 (9)	Cd1^ii^—O8—H8A	112.7
O9—Cd2—O2^ii^	89.88 (12)	Cd1—O8—H8A	112.7
O7^iv^—Cd2—O2^ii^	84.82 (9)	Cd2—O9—Cd1^ii^	121.45 (15)
O2—Cd2—O2^ii^	169.42 (18)	Cd2—O9—Cd1	121.45 (15)
O8^iii^—Cd2—O6^iii^	92.24 (18)	Cd1^ii^—O9—Cd1	94.41 (17)
O9—Cd2—O6^iii^	178.69 (19)	Cd2—O9—H9A	87.8
O7^iv^—Cd2—O6^iii^	78.44 (18)	Cd1^ii^—O9—H9A	117.0
O2—Cd2—O6^iii^	90.00 (12)	Cd1—O9—H9A	117.0
O2^ii^—Cd2—O6^iii^	90.00 (12)	Cd1—O10—H10A	118.4
O8^iii^—Cd2—Cd1^v^	30.80 (7)	Cd1—O10—H10B	103.5
O9—Cd2—Cd1^v^	105.18 (11)	H10A—O10—H10B	109.5
O7^iv^—Cd2—Cd1^v^	143.41 (9)	H1A—N1—H1B	109.5
O2—Cd2—Cd1^v^	69.55 (10)	H1A—N1—H1C	109.5
O2^ii^—Cd2—Cd1^v^	120.64 (10)	H1B—N1—H1C	109.5
O6^iii^—Cd2—Cd1^v^	76.00 (12)	H1A—N1—H1D	109.5
O8^iii^—Cd2—Cd1^iii^	30.80 (7)	H1B—N1—H1D	109.5
O9—Cd2—Cd1^iii^	105.18 (11)	H1C—N1—H1D	109.5
O7^iv^—Cd2—Cd1^iii^	143.41 (9)		
			
O3—S1—O1—Cd1	105.4 (5)	O5—S2—O7—Cd2^vii^	121.6 (4)
O4—S1—O1—Cd1	−134.6 (5)	O1—Cd1—O8—Cd2^vi^	72.2 (6)
O2—S1—O1—Cd1	−13.6 (6)	O9—Cd1—O8—Cd2^vi^	150.2 (2)
O8—Cd1—O1—S1	106.0 (6)	O10—Cd1—O8—Cd2^vi^	−123.5 (2)
O9—Cd1—O1—S1	30.2 (6)	O4^i^—Cd1—O8—Cd2^vi^	−34.0 (2)
O10—Cd1—O1—S1	−58.5 (6)	O5—Cd1—O8—Cd2^vi^	50.0 (3)
O4^i^—Cd1—O1—S1	−146.0 (6)	Cd1^ii^—Cd1—O8—Cd2^vi^	127.1 (3)
O5—Cd1—O1—S1	128.5 (6)	O1—Cd1—O8—Cd1^ii^	−54.8 (6)
Cd1^ii^—Cd1—O1—S1	62.5 (6)	O9—Cd1—O8—Cd1^ii^	23.12 (17)
Cd2^vi^—Cd1—O1—S1	156.8 (4)	O10—Cd1—O8—Cd1^ii^	109.44 (16)
O3—S1—O2—Cd2	−171.0 (3)	O4^i^—Cd1—O8—Cd1^ii^	−161.09 (15)
O1—S1—O2—Cd2	−50.3 (4)	O5—Cd1—O8—Cd1^ii^	−77.1 (2)
O4—S1—O2—Cd2	70.2 (4)	Cd2^vi^—Cd1—O8—Cd1^ii^	−127.1 (3)
O8^iii^—Cd2—O2—S1	−18.0 (4)	O8^iii^—Cd2—O9—Cd1^ii^	−59.33 (18)
O9—Cd2—O2—S1	71.1 (4)	O7^iv^—Cd2—O9—Cd1^ii^	120.67 (18)
O7^iv^—Cd2—O2—S1	171.4 (4)	O2—Cd2—O9—Cd1^ii^	−154.6 (2)
O2^ii^—Cd2—O2—S1	159.8 (9)	O2^ii^—Cd2—O9—Cd1^ii^	36.0 (2)
O6^iii^—Cd2—O2—S1	−110.2 (4)	Cd1^v^—Cd2—O9—Cd1^ii^	−85.89 (17)
Cd1^v^—Cd2—O2—S1	−35.2 (3)	Cd1^iii^—Cd2—O9—Cd1^ii^	−32.8 (2)
Cd1^iii^—Cd2—O2—S1	−36.5 (4)	O8^iii^—Cd2—O9—Cd1	59.33 (18)
O3—S1—O4—Cd1^v^	175.6 (3)	O7^iv^—Cd2—O9—Cd1	−120.67 (18)
O1—S1—O4—Cd1^v^	54.5 (3)	O2—Cd2—O9—Cd1	−36.0 (2)
O2—S1—O4—Cd1^v^	−66.0 (3)	O2^ii^—Cd2—O9—Cd1	154.6 (2)
O6—S2—O5—Cd1	−77.9 (6)	Cd1^v^—Cd2—O9—Cd1	32.8 (2)
O5^ii^—S2—O5—Cd1	44.7 (8)	Cd1^iii^—Cd2—O9—Cd1	85.89 (17)
O7—S2—O5—Cd1	161.1 (5)	O1—Cd1—O9—Cd2	9.6 (2)
O1—Cd1—O5—S2	−156.4 (6)	O8—Cd1—O9—Cd2	−154.3 (2)
O8—Cd1—O5—S2	17.4 (6)	O10—Cd1—O9—Cd2	104.0 (2)
O9—Cd1—O5—S2	−62.2 (6)	O5—Cd1—O9—Cd2	−70.9 (2)
O4^i^—Cd1—O5—S2	116.8 (6)	Cd1^ii^—Cd1—O9—Cd2	−131.3 (3)
Cd1^ii^—Cd1—O5—S2	−24.9 (5)	Cd2^vi^—Cd1—O9—Cd2	−139.10 (15)
Cd2^vi^—Cd1—O5—S2	42.3 (5)	O1—Cd1—O9—Cd1^ii^	140.89 (19)
O5^ii^—S2—O6—Cd2^vi^	−60.4 (3)	O8—Cd1—O9—Cd1^ii^	−22.97 (17)
O5—S2—O6—Cd2^vi^	60.4 (3)	O10—Cd1—O9—Cd1^ii^	−124.63 (17)
O7—S2—O6—Cd2^vi^	180.0	O5—Cd1—O9—Cd1^ii^	60.45 (19)
O6—S2—O7—Cd2^vii^	0.000 (2)	Cd2^vi^—Cd1—O9—Cd1^ii^	−7.76 (18)
O5^ii^—S2—O7—Cd2^vii^	−121.6 (4)		

Symmetry codes: (i) *x*, −*y*+1, *z*−1/2; (ii) −*x*+1, *y*, *z*; (iii) −*x*+1, −*y*+1, *z*+1/2; (iv) *x*, *y*−1, *z*; (v) *x*, −*y*+1, *z*+1/2; (vi) −*x*+1, −*y*+1, *z*−1/2; (vii) *x*, *y*+1, *z*.

### Hydrogen-bond geometry (Å, °)

**Table:**

*D*—H···*A*	*D*—H	H···*A*	*D*···*A*	*D*—H···*A*
O10—H10B···O7^vi^	0.85	2.25	3.087 (5)	167
O10—H10B···O5^i^	0.85	2.36	2.985 (6)	131
O9—H9A···O6^iv^	0.85	2.18	3.029 (7)	173
O8—H8A···O5^i^	0.85	2.56	3.322 (9)	150
O8—H8A···O5^vi^	0.85	2.56	3.322 (9)	150
N1—H1B···O10^vii^	0.90	2.26	2.948 (6)	133
N1—H1D···O4^i^	0.90	2.18	3.077 (5)	180
N1—H1C···O4^viii^	0.90	2.19	3.074 (7)	168
N1—H1A···O3^ix^	0.90	2.38	2.995 (6)	126
N1—H1D···O2^i^	0.90	2.64	3.196 (7)	121

Symmetry codes: (vi) −*x*+1, −*y*+1, *z*−1/2; (i) *x*, −*y*+1, *z*−1/2; (iv) *x*, *y*−1, *z*; (vii) *x*, *y*+1, *z*; (viii) −*x*+3/2, −*y*+3/2, *z*−1/2; (ix) −*x*+3/2, *y*+1/2, *z*.
